# A Stretchable Pressure-Sensitive Array Based on Polymer Matrix

**DOI:** 10.3390/s17071571

**Published:** 2017-07-05

**Authors:** Yuanzheng Luo, Qi Xiao, Buyin Li

**Affiliations:** School of Optical and Electronic Information, Huazhong University of Science and Technology, Wuhan 430074, China; D201377509@hust.edu.cn (Y.L.); M201572111@hust.edu.cn (Q.X.)

**Keywords:** sensor array, graphene foam, piezo-resistivity, PDMS

## Abstract

Herein, a flexible 6 × 6 pressure-sensitive array (based on the PDMS (Polydimethylsiloxane) porous substrate) was designed. We have developed a facile method to fabricate the porous substrate, by a single-step operation using the sugar-template method. This strategy effectively diminishes the complexity of the preparation process, as well as the device structure. The electrical resistivity of the stretchable array demonstrates the negative piezo resistive coefficient (NPRC) under 0–100 kpa. Moreover, the pressure-sensitive array reveals a high sensitivity and low delay time (<0.5 s) to the applied forces. Therefore, the pressure distribution could be easily recognized by testing its conductivity changes. Besides, these signal data can be collected into the upper computer, with the purpose of tracking and analyzing the azimuth of the applied loading. This cost-effective micro array has a broad application prospect for fabricating the tactile sensor, artificial skin, and human-computer interfaces.

## 1. Introduction

After years of development, mechanical sensors made up of elastomeric rubber and nano-sized conductive filler have been exploited as candidates, so as to fabricate flexible, resistive-type mechanical sensors. Due to the ease and low-cost of fabrication, as well as the simple signal conversion and readout mechanism, resistive-type mechanical sensors have been widely employed for the purpose of realizing cost-effective, wearable electronics. In particular, composites with graphene-supported polymer coating have been proved to have great potential in flexible electronics, such as flexible pressure sensors [1], artificial skin [2], and wearable electronics [3], for the reason that they only require two components: piezo-resistive material and an electrode. However, even if these explorations of functional materials for sensing applications have made some achievements, the fabrication of the pressure sensor arrays towards practical applications is still a challenging task. Besides, the sensitivity of the pressure sensors is one of the most essential factors for better capturing human motions and emulating human skin [4]. There are two effective methods to achieve high sensitivity: utilizing the sensitive op-amp input transistors to amplify signals, and measuring strain changes in the capacitance. Nevertheless, these methods require a complicated microfabrication structure [5,6]. Thus, another facile approach to enhance sensitivity is to introduce porous microstructures into the conductive nanomaterials [7]. Based on present reports, most research developed the soft or porous sensitive elements on the rigid polymer substrate. These rubber-like sensor structures could only measure local deformations with poor flexibility and sensitivity, which limits their further application. In terms of the highly sensitive sensor array, the porous foam-like microstructures are equally important to the piezo-resistive material and the substrate material.

PDMS (Polydimethylsiloxane) is a biocompatible elastomer, which can be easily integrated with human bodies, as well as the flexible and stretchable sensor device; therefore, the PDMS substrate is widely used in the mechanical piezo-resistive sensors [8,9]. Furthermore, Cheng et al. [10] have fabricated 8 × 8 stretchable tactile sensing arrays, and the PDMS has been employed both for the fabrication of the skin structure and effective encapsulation. Mei et al. [11] have designed a pressure-sensitive 4 × 4 array on the basis of the conductive rubber. Besides, the base material is silicone rubber, and the delay time is less than 2 s under an applied loading from 0 N to 4 N. Kazemzadeh et al. [12] have also fabricated a graphene-based sensor array by using the laser scribing method, which refers to the highly sensitive sensor elements that are not integrated into any soft substrate. These fabricated arrays are only adaptable for some applications with a low delay time and small measurement range. In general, the rigid and fragile substrate of pressure-sensitive array will bring about failures on the permissible stress, or repeated deformation [13]. At the same time, their manufacturing process is quite complex, and a homogeneous polymer substrate will be gained only through chemical cross-linking. Owing to the different phases between porous sensing units and the rigid package substrate, the superior electromechanical properties of nano-sized, filled, conductive material would be restrained by the incompatibility. Porous package is desirable for highly sensitive sensors because it does not restrain the intrinsic performance or the structural component during actuation; furthermore, the porous PDMS substrate is preferred. For one thing, recently, the exploration of graphene foams is directly motivated by its possibility for potential applications, such as light weight polyurethane (PU)/graphene sensor [14] and highly sensitive PDMS/graphene sensor [1]. Especially, Yao et al. [15] have designed a microstructure graphene foam through simple solution dip-coating, and as a result, the sensor has high sensitivity and cycling stability for the artificial skin.

As stated above, the high-performance sensor array should have a consistently built porous structure of both sensing units and substrate material. Herein, the sugar-templated method, a low-cost and environmentally benign method, is used to yield a hierarchically porous PDMS substrate. Meanwhile, the graphene-based polymer dip-coating, a feasible and scalable strategy, is introduced to improve the electromechanical properties of commercial PU foam. As sensing units, the graphene-coated foams (GCF) are integrated in PDMS porous substrate to develop a light-weight, stretchable, and durable array, for the purpose of monitoring the applied pressure and maintaining excellent electrical conductivity under a high mechanical stress. After assembling the GCF sensor elements into the PDMS matrix, a flexible pressure-sensitive sensor with high-sensitivity can be obtained. Moreover, a test circuit was designed to measure the pressure on the sensor array.

## 2. Materials and Methods

### 2.1. The Structure of the Pressure-Sensitive Array

**Figure 1:**
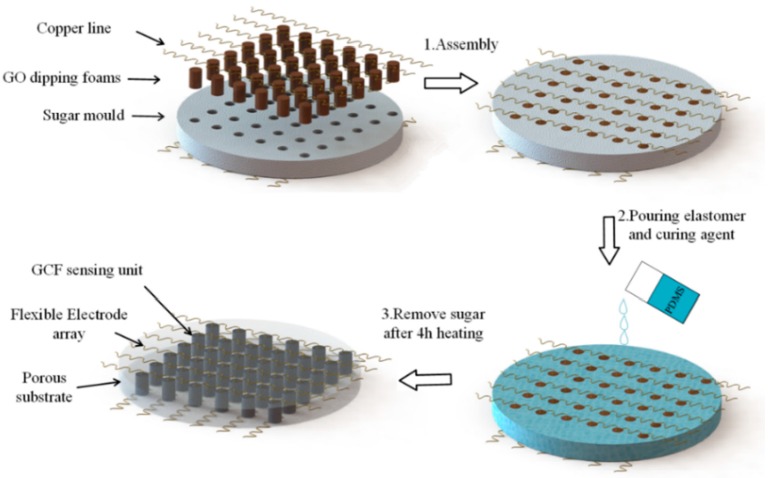
The manufacturing process of the porous substrate and the schematic diagram of the pressure-sensitive array.

**Figure 2:**
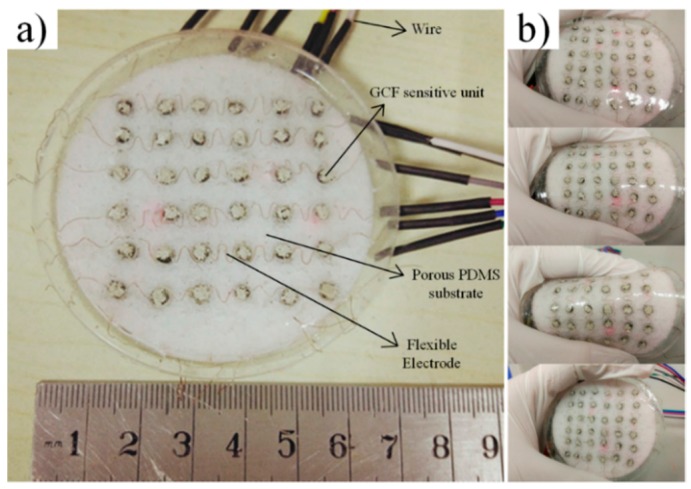
(**a**) A digital photograph of the prepared pressure-sensitive array; (**b**) the stretchable sensor array exhibits large flexibility.

The schematic of the fabrication process of the sensor array is demonstrated in Figure 1. The fabrication includes the following three facile steps: (1) assembling the cylindrical graphene oxide (GO)/foams into the holes of the sugar mould and attaching the copper lines into its surface; (2) pouring the liquid polymer in the sugar template (as a two-part polymer, base elastomer and curing agent (PDMS) is used); (3) Curing the base material and reducing the GO dipping element in a single heat step, and taking off the sugar by water dissolving. The hierarchical structure of the pressure-sensitive array consists of the upper electrodes, the pressure sensitive GCF, the insulating porous substrate, and the bottom electrodes. First, the upper and bottom electrodes are soft, and the flexible copper wires are attachable to the surface of the base material. Second, the curved shape is obtained by manually twisting, and the contact area of the sensor elements is consolidated using low temperature silver paste. Moreover, there are corresponding through-holes in the sugar mould, to locate and fix the sensing units. The number and size of through-holes have matched the cylindrical GCF. In addition, the insulating porous substrate guarantees that the large deformation of the sensing elements is in accordance with the whole package device. In the horizontal plane, the bottom and upper electrode arrays are placed orthogonally, and this spread electron array and horizontal-to-vertical configuration maintain a large proportion of the active sensing area. Furthermore, the thickness of the electrodes and pressure sensitivity rubber is 0.1 mm and 4 mm, respectively. The thickness of the total pressure-sensitive array is 10 mm, and the radius is 35 mm, as demonstrated in Figure 2a.

### 2.2. The Fabrication of the Sensor Array

The sugar mould was fabricated by mixing fine granulated sugar and water at a ratio of 35:1, and directly followed by a drying procedure in an air-circulating oven at 90 °C for 30 min. We tried various granulated sugars with different granularity, such as coarse sugar, sanding sugar, and regular white sugar. In addition to these large crystal sugars, castor sugar with ultrafine granularity is ideal for sugar template formulation since it dissolves easily and smooth crystal could be more homogeneous. This is probably due to the fact that the size of the sugar particles in the templates were notably different [16]. Furthermore, the homogeneous and compact sugar mould can withstand reasonable handling and machining without collapse; the 6 × 6 through-hole array can be formed by drill-hole directly. The manufacturing procedure of the PDMS matrix is demonstrated in Figure 1, and the sugar, with ultrafine granularity, was used to form the sacrificial template with a well-connected microporous. The production of GCF was performed through the simple solution dip-coating of GO on the commercial polyurethane (PU) sponges (3M Company, St. Paul, MN, USA). The GO aqueous solution of 4 mg/mL was purchased from Graphenea Inc (San Sebastián, Spain). The tailored PU sponges were first dip-coated in the GO suspension by a repetitive squeezing, and then placed in the through-hole array of the sugar template; the GO was reduced finally by the step mentioned below, at 120 °C for 4 h. The reduction process could induce the self-assembly of the reduced graphene oxide (rGO) sheets, and lead to the formation of the graphene-coated structure in GCF. By using the direct template method, we have performed a package of the prepared copper wires and GO/foams in this procedure. The liquid PDMS (Sylgard 184, Dow Corning (China) Holding Ltd., Beijing, China) was prepared by mixing the base elastomer and curing agent at a 10:1 weight ratio. The obtained porous substrate with this ratio can possess a relatively higher strength and durability, as previously reported [17]. After pouring it straightly into the culture dishes and being immersed with the above integrated products, it was placed in a vacuum oven about 45 min, to fully exhaust the air bubbles; subsequently, it was heated at 120 °C for 4 h to solidify the mixture. Then, the resultant was soaked in water for 1 h to dissolve the sugar particles in water. After drying for another hour, the nested graphene-coated foam became the inner packaged sensor element, and the flexible electrode became consistent with the surface layer, which led to the formation of the flexible sensitive sensor array. For the other, the graphene-assembled monoliths (GM) were also prepared for the comparison of surface appearance that followed. 500 μL GO solution was added in a micro test tube, and was reduced at 120 °C for 4 h in sealed state. Then, the resultant was directly dried in an air-circulating oven at 90 °C. During the drying process, the evaporation of water led to the self-assembly of GO sheets, resulting in the formation of a graphene monolith.

### 2.3. Experimental Tests and Characterizations

**Figure 3:**
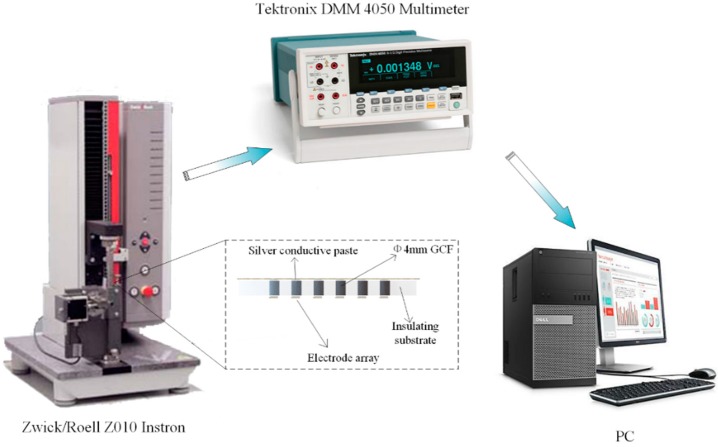
The measurement system.

The piezo-resistive characteristic has been researched with a GCF sample (4 mm diameter and 2 mm height). Electrical conductivity was measured using a two-point probe method. An instron (Zwick/Roell z010, Zwick, Ulm, Germany) that uses a strain control mode with a compressive strain rate of 100% min^−1^ was used to provide the required pressure. Furthermore, a digital multimeter (Tektronix DMM 4050, Tektronix, Shanghai, China) was used to monitor the resistance change of the GCF, which is described in Figure 3. Moreover, the microstructure and morphology was observed by a FEI Quanta 200 (FEI, Hillsboro, OR, USA) Scanning Electron Microscope (SEM) at 20 kV. 

### 2.4. Sensing Systems

**Figure 4:**
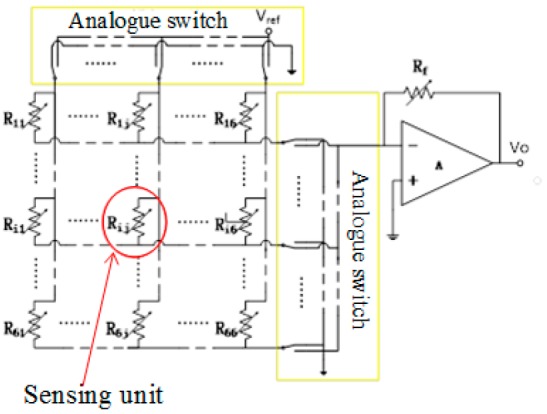
Readout circuit schematic and 6 × 6 example sensor array are presented.

**Figure 5:**
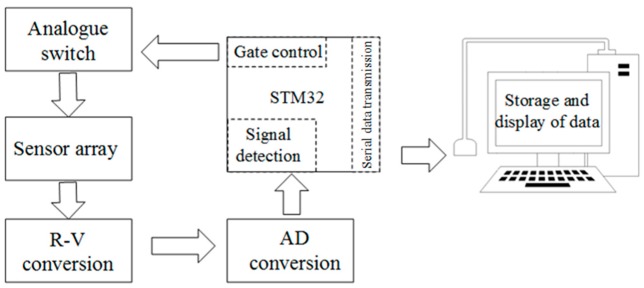
Block diagram of the data acquisition method.

The arrangement of GCF is assembled as a two-dimensional (2D) resistive sensor array to reduce the crosstalk problem [18]. The electrical connection of electrode array utilized the shared row and column connections to simplify the electric connect complexity. All the row-column nodes can be regarded as 36 individual sugar pressure sensing units, as demonstrated in Figure 4. The detection orders of the sensing array are controlled by the four-channel and double-throw analog switches, with the combinations of rows and columns, the scanning module, and the voltage feedback to determine whether the sensing units is pressured or not. We can scan the whole array by switching these analog multiplexers, which are digitally-controlled by the STM32 microcontroller. The microcontroller is controlled only by one independent sensor in the power supply at a time, and its detectable resistance change will be readout sequentially. In addition, Figure 5 exhibits the schematic of the sensing systems. The readout resistance signal could be obtained and converted into the voltage signal on the reference resistance of R_f_ by the R-V conversion circuit. Straight after the analog-to-digital (AD) conversion, the digital voltage signals are sent to PC through the serial port. The further storage and display of the data in real-time can be carried out by the PC software.

## 3. Results

### 3.1. Morphological Analyses of GCF

**Figure 6:**
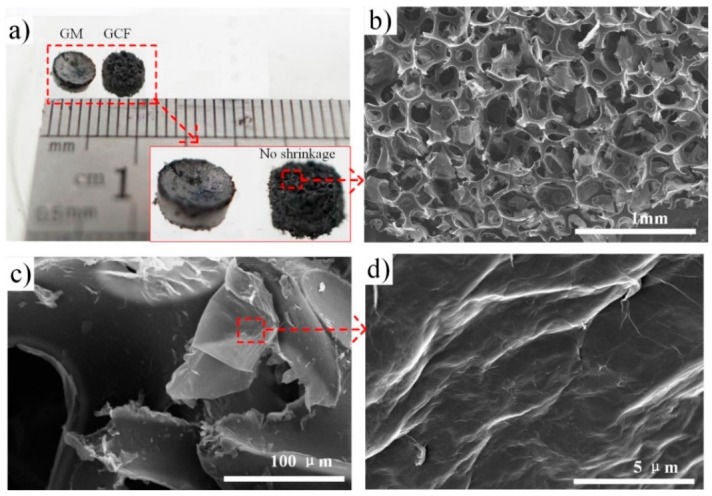
(**a**) Macro photo of the mini size of graphene-coated foams (GCF) and graphene-assembled monoliths (GM) that are synthesized by the same process. The insert that demonstrates GCF can be fabricated with a small size and the well-defined appearance of geometries; (**b**–**d**) show the Scanning Electron Micrscope (SEM) images of GCF under some different resolutions.

The GCF sample has merits over the additive-free GM, especially when it is used as the diminutive sensing elements. Its unique porous microstructure and the function of coated graphene PU backbone give rise to a well-defined morphology in small dimension. The pony-size GCF and GM samples that are obtained through the similar processes (as mentioned above) have different macroscopic shapes; the contrast is indicated in Figure 6a. The bowl-like surface of GM is fragile under stress, and has poor efficiency when contacting the piezo-resistive area. Even if GM serve as promising candidates for strain sensing, owing to their special porous structure, the capillary pressure that is caused by the small porosity will lead to a severe shrinkage during the ambient pressure drying, in accordance with the following equation:

P=(−2γ cosθ)/r

The capillary pressure is associated with the contact angle (*θ*), and the surface tension of the pore liquid (*γ*) is inversely proportional to the pore radius (*r*). Therefore, the larger pore radius of PU skeletons in GCF, demonstrated in Figure 6b, has been effectively minimizing the capillary influence, which is in favor of realizing the low-cost ambient drying, and leads to a well-defined appearance. It has been demonstrated that the small sensitive elements could be easily fabricated and fit the cylindrical through-hole in the substrate. Besides, SEM observation also indicates that the reduced GO sheets are coated on the interconnected PU skeletons (Figure 6c). Moreover, the pristine PU foam has a highly porous structure, with pore sizes of around hundreds of microns [19]. The overall morphology of the GCF has changed little, and the frameworks that cover the transparent graphene sheets have suggested successful assembly on the skeleton. The electrical conductivities of CGF can reach ∼0.1 to 17.8 S/m, because of this three-dimensional (3D) conductive graphene network. In addition to improved electrical properties, the highly compressible PU skeleton has facilitated the mechanical property of GCF. As a strain sensing unit, the GCF can be compressed up to 90% of its original height, and then fully recovered without permanent deformation. These advantages may endow the GCF with an adjustable piezo-resistive behavior in a sensitive array.

### 3.2. Piezo-Resistive Characteristic

**Figure 7:**
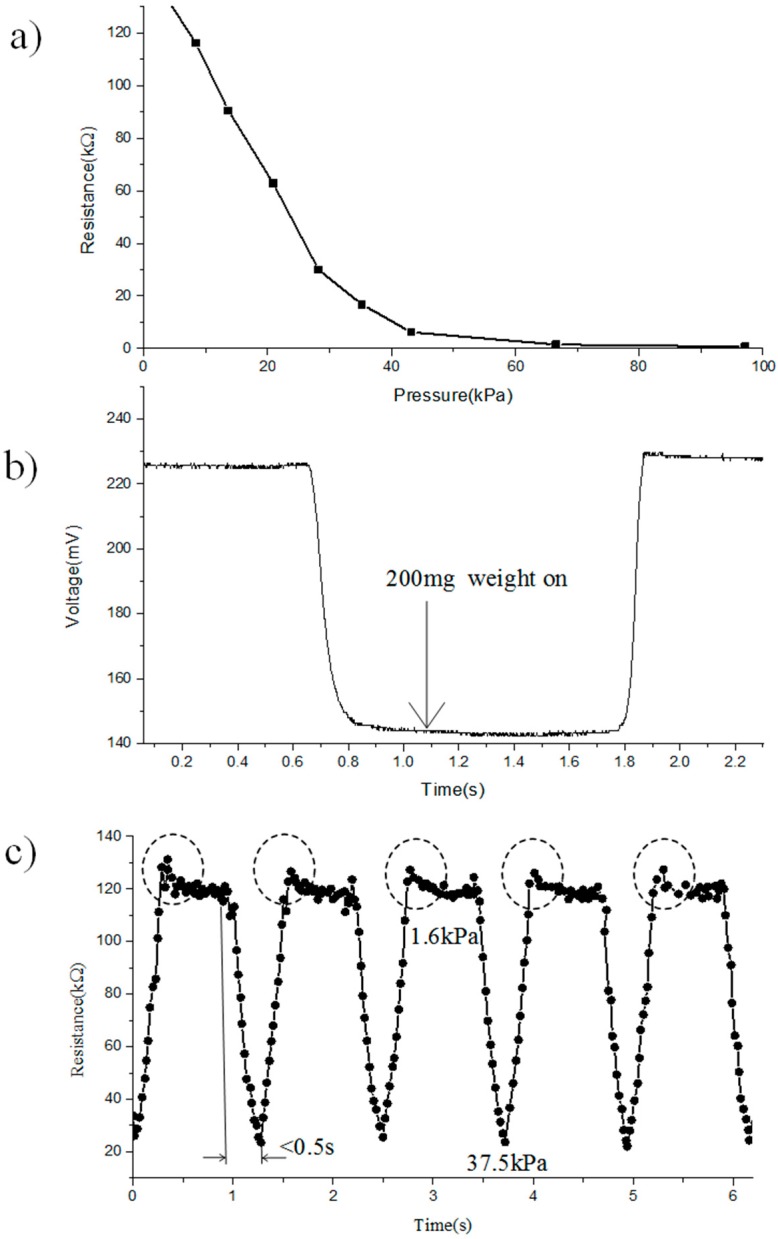
The piezo-resistive characteristic of GCF: (**a**) the relationships between the resistance and applied loading; (**b**) an example of the GCF with the detection of ultralow pressure (200 mg weight on an area of 12.5 mm^2^); (**c**) stability and repeatability.

Figure 7a demonstrates the relationship between the electrical resistance of the sensing unit and the applied loading. When the external forces that are applied on the sensor array have increased from 0 to 95 kPa, the resistance of the sensing units becomes smaller gradually, and as a result, changes from 121.9 kΩ to 56 Ω, as shown in Figure 7a. We have cycled that process several times on each sensing unit, and took the average of these negative pressure coefficient of resistances. Furthermore, Figure 7b indicates an example of the sensor array, detecting the low weight (200 mg weight on an area of 12.56 mm^2^ is equivalent to 1.6 kPa). The pressure sensitivity of GCF is 51 mV/kPa, which is superior to the previously reported rGO sensor array(19 mV/kPa) [12], MEMS sensors (37.5 mV/kPa) [20], and carbon nanotubes (CNT)-based sensor (10 mV/kPa) [5]. Figure 7c shows the robustness and recyclability of the pressure-sensitive array. The sensing unit has demonstrated a constant response time when increasing applied loading from 1.6 kPa to 37.5 kpa. The general response time is less than 0.5 s, which is much faster than the reported sensor array, which has been encapsulated into the rigid PDMS substrate [11]. When the applied loading has releases quickly, it also indicates a similar trend across the unloading process. Moreover, there is a smaller pulse resistance than the commercial conductive rubber [11], just as circled in Figure 7c. The reason for the unique property is the fast relaxation deformation process of the sensor array, which has combined both the robust porous substrate encapsulation and high sensitivity porous material. In practice, with its stress-dependent resistance to detect the loading force reversibly, and the combination of the porous sensing elements and substrate, as well as the low delay, all these have indicated the possible application of real-time force detection with high sensitivity.

### 3.3. Piezo-Resistive Mechanism of GCF

**Figure 8:**
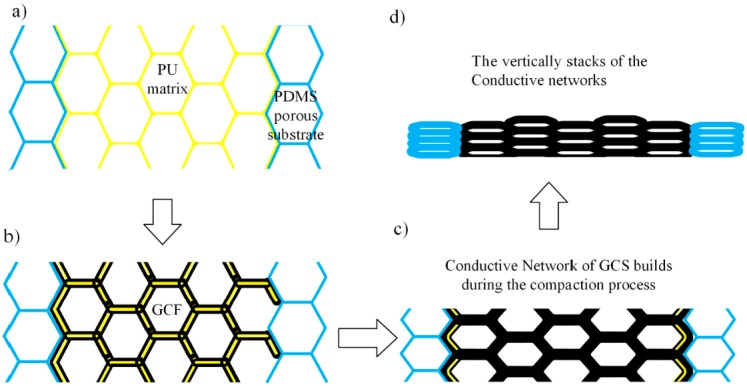
The piezo-resistive mechanism is in the composite: (**a**) interconnected network of the polyurethane (PU) matrix; (**b**) initial conductive paths are being built, owing to the graphene sheets coating; (**c**) the resistance of the conductive path has been enhanced, due to the increased density of graphene sheets; (**d**) the stacks of the conductive path until the compression limit.

Even if the variation of electrical resistance arises from the deformation and loading force, the sensing mechanism of 3D graphene foam is mainly established on the change of the conductive networks. Unlike carbon nanotubes/silicone rubber nanocomposites, the GCF has an initial electrical conductivity owing to the interconnected PU matrix with graphene-coated “skin”, as shown in Figure 8a,b. After the abovementioned graphene-coated process, the interconnected conductive paths are formed with the presence of graphene skin, as described in Figure 8b. Together with the developed porous substrate, light contact pressure could induce the increase of initial conductive pathways contributes to the output signal in the low-pressure sensing process. Owing to this elastic and conductive GCF matrix, higher compressive strain/stress refers to a better contact between the graphene sheets, which brings about a higher density of graphene sheets, and at the same time, a greater electrical conductivity of composite. The conductive path through the GCF is controlled by the bulkier graphene sheets-wrapped matrix, as shown in Figure 8c. Together with the vertical stack of conductive networks, the void space of the GCF skeleton has been reduced to the limit, and the graphene sheets that are conducting the path are of primary importance, and play a major role in guaranteeing the excellent conductivity, as shown in Figure 8d. Additionally, because the continuous graphene-coated PU branches have an excellent elasticity and changeable conductive path, the GCF reveals a reversible and immediate NPRC effect.

### 3.4. Measurement and Results

**Figure 9:**
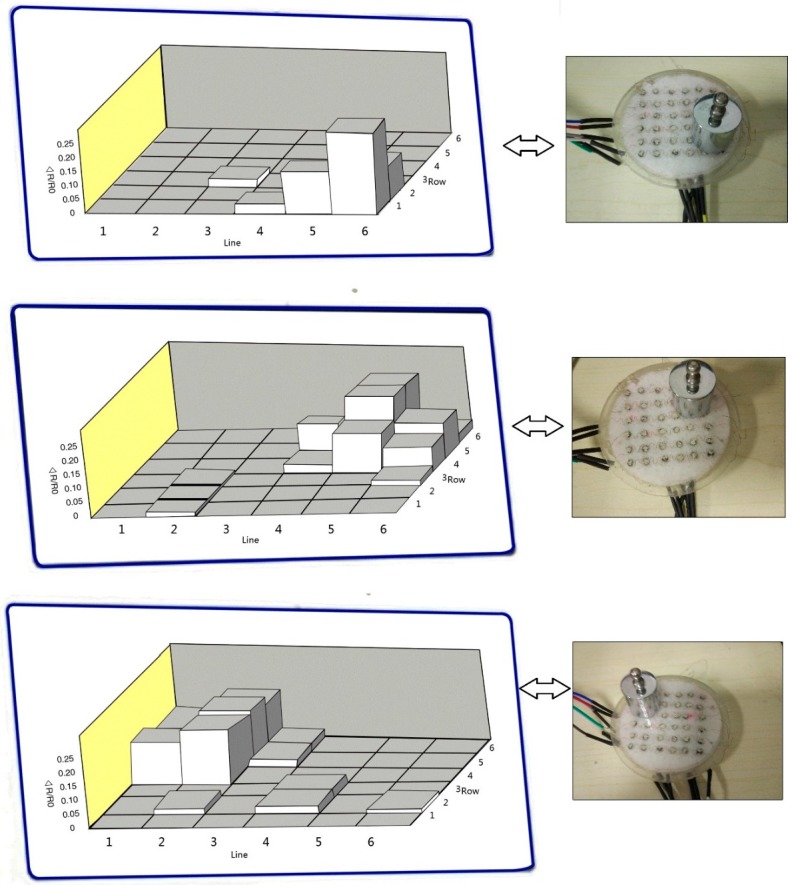
A sensor array with the weight placed on the surface to detect the different identifiable locations. The 100 g cylindrical standard weight was used to provide applied loading in the testing, shown in the inset.

**Figure 10:**
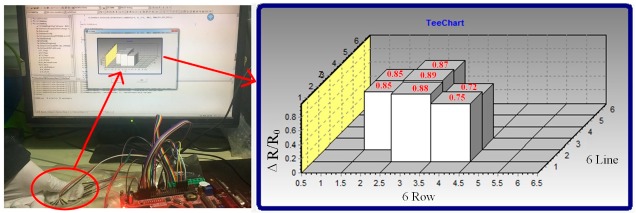
The test process (the red circle is the manually applied pressure on the array). It brings about three-dimensional displays.

For tactile sensing and human-computer interfaces, the pressure sensor array needs to be developed to detect pressure distribution. The position of the weight could be identified by testing their conductivity changes in our sensing system. Figure 9 shows simple tests, realized by placing different positions of the weight to detect pressure distribution. The area of each sensing unit is 12.5 mm^2^. The 100 g cylindrical standard weight was used to provide applied loading in the testing. Output voltages of the sensing elements can be transferred to computer software for analysis. The position obtained from the pressure sensor array is consistent with the positions. Figure 10 demonstrates a pressure test to observe the response of the sensor array under the compress strain. Quasi-static pressure is applied by using the thumb to manually press the sensor array surface. The prominence thumb presses three sensing units, and then the side of the thumb contacts the other four elements until the equilibrium conditions appear. The response reveals a good spatial localization that is presented as a three-dimensional histogram, which also conforms to the application of the Quasi-static pressure area on the array. Meanwhile, the column height (the value marked in red) is used to display the resistance change (ΔR/R_0_) values. In accordance with the relationships between the resistance and applied loading, the amount of pressure can be visually recognized as well. Besides, the data disparity of the column height can explain the inhomogeneous contact force on the array surface, because the thumb prominence is spherical. Owing to the high spatial resolution and sensitivity of the 6 × 6 sensor array, it is very easy to exactly define the place of the applied pressure and its degree. Additionally, the uncertainty of the large sensor array commonly results from crosstalk effect, and instrumental bias errors were also observed. In order to assess these errors, a number of readings (n = 10 times) are performed for each sensing position. The measurement errors that occur in actual sensor data reveal a strong positive correlation with increased external loading, as shown in Table 1. The subscript of S*ij* is in accordance with the R*ij* in Figure 5, and we take regional dates in S_11_–S_33_ as typical to avoid lengthy data. The maximum deviation is less than 10%; also indicated is good repeatability and compatibility between the sensing units and porous substrate at the core sensing regions.

Recently, a lot of pressure sensor arrays have been developed based on different principles, such as capacitive and piezoresistive. Some of these previously reported works, classified on the basis of sensitivity, range of pressure, functional material, and substrate, are compared to our study in Table 2. In comparison, our array made of 6 × 6 elements possesses higher sensitivity than the similar piezoresistive arrays in a wide pressure range. The response-time of the traditional piezoresistive sensors is slow due to the sluggish relaxation and hysteresis effect of the polymer substrate, especially compared to the capacitance sensor. Our sensor array with the cellular structure substrate for fast response is close to some capacitive arrays, due to its flexibility and fast stress relaxation, in both porous units and substrate. The substrate-dependent sensing units can be highly sensitive and the sensor arrays are capable of giving a fast response.

## 4. Conclusions

To sum up, by combining the high conductivity of the graphene-coated foam elements, as well as the high compressibility of the porous PDMS substrate, we successfully fabricated a flexible and stretch strain sensor array for the purpose of piezo-resistive and spatial sensing. The preparation process of GCF is made at a low temperature (<100 °C) and ambient pressure. The fabrication of the porous substrate structure using a sugar-template method is also facile and clean. Notably, the designed porous package structure can effectively reduce the response-recovery delay, and the complexity of the device. Through the real-time monitoring system, the pressure-sensitivity array demonstrates 200 mg weight sensitivity and less than a 0.5 s delay time, to both loading and unloading. Moreover, the sensor array measurement possessed a high spatial resolution of the applied touch pressure, and the amount of pressure can also be visually recognized. Due to its low cost and ease of preparation, instantaneous response, and flexibility, the sensor array can be implemented as a sensor device, useful for applications in different fields from wearable equipment to automotive and aerospace engineering. Thus, the findings in this work will contribute to the realization of large-scale and cost-effective commercial production of the high-performance sensor array.

## Figures and Tables

**Table 1:** The bias error for the sensing units in each horizontal-vertical channel are recorded.

Actual Pressure = 10 N			Actual Pressure = 50 N		
Sensing Position	Average Experimental Values (N)	Error (%)	Sensing Position	Average Experimental Values (N)	Error (%)
S_11_	10.6	1.2	S_11_	45.8	8.4
S_12_	10.2	0.4	S_12_	46.4	7.2
S_13_	11.4	2.8	S_13_	47.6	5.6
S_21_	11.8	3.6	S_21_	45.9	8.2
S_22_	11.5	3.0	S_22_	46.1	7.8
S_23_	11.7	3.4	S_23_	46.7	6.6
S_31_	12.4	4.8	S_31_	45.1	9.8
S_32_	11.6	3.2	S_32_	46.6	6.8
S_33_	10.8	1.6	S_33_	47.1	5.8

**Table 2:** Comparison of similar sensor arrays, previously reported.

Ref.	Functional Material	No. of Sensing Units	Substrat	Sensor Type	Range	Responses	Sensitivity
[8]	CNTs	9	PDMS	Capacitance	<10 kPa	200 ms	19.8% kPa^−1^
[12]	rGO	5	—	Piezoresistive	—	1 s	19 mV/kPa
[11]	Conductive rubber	16	PDMS	Piezoresistive	0–320 kPa	2 s	—
Our work	GCF	36	Porous PDMS	Piezoresistive	0–95 kPa	500 ms	51 mV/kPa

## Abbreviations

The following abbreviations are used in this manuscript:

PDMS	Polydimethylsiloxane
NPRC	Negative piezo resistive coefficient
GCF	Graphene-coated foam
rGO	Reduced graphene oxide
GO	Graphene oxide
3D	Three-dimensional
2D	Two-dimensional
CNT	Carbon nanotube
PU	Polyurethane
SEM	Scanning electron microscopy

## References

[1] Rinaldi A., Tamburrano A., Fortunato M., Sarto M. (2016). A Flexible and Highly Sensitive Pressure Sensor Based on a PDMS Foam Coated with Graphene Nanoplatelets. Sensors.

[2] Yang Y.J., Cheng M.Y., Chang W.Y., Tsao L.C., Yang S.A., Shih W.P., Chang F.Y., Chang S.H., Fan K.C. (2008). An integrated flexible temperature and tactile sensing array using PI-copper films. Sens. Actuators A Phys..

[3] Lim S., Son D., Kim J., Lee Y.B., Song J.K., Choi S., Lee D.J., Kim J.H., Lee M., Hyeon T. (2014). Wearable Electronics: Transparent and Stretchable Interactive Human Machine Interface Based on Patterned Graphene Heterostructures. Adv. Funct. Mater..

[4] Zang Y., Zhang F., Di C., Zhu D. (2014). Advances of flexible pressure sensors toward artificial intelligence and health care applications. Mater. Horiz..

[5] Lipomi D.J., Vosgueritchian M., Tee B.C., Hellstrom S.L., Lee J.A., Fox C.H., Bao Z. (2011). Skin-like pressure and strain sensors based on transparent elastic films of carbon nanotubes. Nat. Nanotechnol..

[6] Schwartz G., Tee C.K., Mei J., Appleton A.L., Kim D.H., Wang H., Bao Z. (2013). Flexible polymer transistors with high pressure sensitivity for application in electronic skin and health monitoring. Nat. Commun..

[7] Jung S., Kim J.H., Kim J., Choi S., Lee J., Park I., Hyeon T., Kim D.H. (2014). Reverse-Micelle-Induced Porous Pressure-Sensitive Rubber for Wearable Human–Machine Interfaces. Adv. Mater..

[8] Cui J., Zhang B., Duan J., Guo H., Tang J. (2016). Flexible Pressure Sensor with Ag Wrinkled Electrodes Based on PDMS Substrate. Sensors.

[9] Hasan S.A.U., Jung Y., Kim S., Jung C.L., Oh S., Kim J., Lim H. (2016). A Sensitivity Enhanced MWCNT/PDMS Tactile Sensor Using Micropillars and Low Energy Ar^+^ Ion Beam Treatment. Sensors.

[10] Cheng M.Y., Tsao C.M., Lai Y.Z., Yang Y.J. (2011). The development of a highly twistable tactile sensing array with stretchable helical electrodes. Sens. Actuators A Phys..

[11] Mei H., Wang R., Feng J., Xia Y., Zhang T. (2015). A flexible pressure-sensitive array based on soft substrate. Sens. Actuators A Phys..

[12] Kazemzadeh R., Andersen K., Motha L., Kim W.S. (2015). Highly Sensitive Pressure Sensor Array with Photothermally Reduced Graphene Oxide. IEEE Electron Device Lett..

[13] Stassi S., Cauda V., Canavese G., Pirri C.F. (2014). Flexible Tactile Sensing Based on Piezoresistive Composites: A Review. Sensors.

[14] Liu H., Dong M., Huang W., Gao J., Dai K., Guo J., Zheng G., Liu C., Shen C., Guo Z. (2017). Lightweight conductive graphene/thermoplastic polyurethane foams with ultrahigh compressibility for piezoresistive sensing. J. Mater. Chem. C.

[15] Yao H.B., Ge J., Wang C.F., Wang X., Hu W., Zheng Z.J., Ni Y., Yu S.H. (2013). A flexible and highly pressure-sensitive graphene-polyurethane sponge based on fractured microstructure design. Adv. Mater..

[16] Choi S.J., Kwon T.H., Im H., Moon D.I., Baek D.J., Seol M.L., Duarte J.P., Choi Y.K. (2011). A polydimethylsiloxane (PDMS) sponge for the selective absorption of oil from water. ACS Appl. Mater. Interfaces.

[17] Zhao X., Li L., Li B., Zhang J., Wang A. (2014). Durable superhydrophobic/superoleophilic PDMS sponges and their applications in selective oil absorption and in plugging oil leakages. J. Mater. Chem. A.

[18] Saxena R.S., Bhan R.K., Saini N.K., Muralidharan R. (2011). Virtual Ground Technique for Crosstalk Suppression in Networked Resistive Sensors. IEEE Sens. J..

[19] Shen B., Yang L., Zhai W., Zheng W. (2016). Compressible Graphene-Coated Polymer Foams with Ultralow Density for Adjustable Electromagnetic Interference (EMI) Shielding. ACS Appl. Mater. Interfaces.

[20] Huang X., Zhang D. (2014). A high sensitivity and high linearity pressure sensor based on a peninsula-structured diaphragm for low-pressure ranges. Sens. Actuators A Phys..

